# S01-3: Developing a new physical activity outcomes framework for Scotland

**DOI:** 10.1093/eurpub/ckae114.199

**Published:** 2024-09-26

**Authors:** Niall Taylor, Jennifer Love

**Affiliations:** Scottish Government; Scottish Government

## Abstract

**Purpose:**

To share learning from the development of a new physical activity outcomes framework as part of the application of a systems-based approach to physical activity policy in Scotland.

**Development:**

The Active Scotland Outcomes Framework first published in 2014 focused on six high level outcomes against which national progress was monitored.

The Public Health Scotland (PHS) systems-based approach to physical activity (PA) proposed the adoption of eight thematic delivery outcomes, prompting the revision of the Active Scotland Outcomes Framework and associated indicators.

This coincided with the development of a Population Health Plan for Scotland and the need to identify a PA target to sit alongside other health improvement targets. Application of the WHO target to reduce the relative global prevalence of physical inactivity in adults and adolescents by 15% by 2030 was identified as the logical target to follow, but to do so it would need to be defined and quantified in a Scottish context.

**Implementation and dissemination:**

Subsequently the revised Active Scotland Outcomes Framework now states a long-term vision underpinned by eight delivery outcomes mapped against associated indicators and aligned to the WHO target to reduce the relative global prevalence of physical inactivity in adults and adolescents by 15% by 2030.

WHO guidance indicates 2016 as the baseline year for this target. In that year 36% of adults and 39% of children in Scotland were classed as inactive (not meeting the recommended levels of MVPA). Therefore, by 2030 a relative reduction in inactivity in Scotland would equate to 30% of adults and 33% of children.

**Evaluation:**

The revised outcomes framework associated indicators and target are used to evaluate the new Physical Activity for Health strategy. Data are largely derived from two annual national surveillance surveys: The Scottish Health Survey and the Scottish Household Survey.

**Conclusions and practical implications:**

Adoption of a systems-based approach derived from international evidence and best practice, translated into a Scottish context provides a robust framework through which a new strategy, outcomes framework and target for Scotland were developed.

